# Global miRNA expression analysis of serous and clear cell ovarian carcinomas identifies differentially expressed miRNAs including miR-200c-3p as a prognostic marker

**DOI:** 10.1186/1471-2407-14-80

**Published:** 2014-02-11

**Authors:** Bente Vilming Elgaaen, Ole Kristoffer Olstad, Kari Bente Foss Haug, Berit Brusletto, Leiv Sandvik, Anne Cathrine Staff, Kaare M Gautvik, Ben Davidson

**Affiliations:** 1 Department of Gynecological Oncology, Oslo University Hospital (OUH), The Norwegian Radium Hospital, Postbox 4953 Nydalen 0424, Oslo, Norway; 2 Department of Medical Biochemistry, OUH, Ullevaal, Oslo, Norway; 3 Faculty of Medicine, University of Oslo, Oslo, Norway; 4 Department of Biostatistics and Epidemiology, OUH, Ullevaal, Oslo, Norway; 5 Department of Gynecology and Obstetrics, OUH, Ullevaal, Oslo, Norway; 6 Department of Pathology, OUH, The Norwegian Radium Hospital, Oslo, Norway

**Keywords:** Ovarian carcinoma, MicroRNA, Microarray, Quantitative PCR, Survival

## Abstract

**Background:**

Improved insight into the molecular characteristics of the different ovarian cancer subgroups is needed for developing a more individualized and optimized treatment regimen. The aim of this study was to a) identify differentially expressed miRNAs in high-grade serous ovarian carcinoma (HGSC), clear cell ovarian carcinoma (CCC) and ovarian surface epithelium (OSE), b) evaluate selected miRNAs for association with clinical parameters including survival and c) map miRNA-mRNA interactions.

**Methods:**

Differences in miRNA expression between HGSC, CCC and OSE were analyzed by global miRNA expression profiling (Affymetrix GeneChip miRNA 2.0 Arrays, n = 12, 9 and 9, respectively), validated by RT-qPCR (n = 35, 19 and 9, respectively), and evaluated for associations with clinical parameters. For HGSC, differentially expressed miRNAs were linked to differentially expressed mRNAs identified previously.

**Results:**

Differentially expressed miRNAs (n = 78) between HGSC, CCC and OSE were identified (FDR < 0.01%), of which 18 were validated (p < 0.01) using RT-qPCR in an extended cohort. Compared with OSE, miR-205-5p was the most overexpressed miRNA in HGSC. miR-200 family members and miR-182-5p were the most overexpressed in HGSC and CCC compared with OSE, whereas miR-383 was the most underexpressed. miR-205-5p and miR-200 members target epithelial-mesenchymal transition (EMT) regulators, apparently being important in tumor progression. miR-509-3-5p, miR-509-5p, miR-509-3p and miR-510 were among the strongest differentiators between HGSC and CCC, all being significantly overexpressed in CCC compared with HGSC. High miR-200c-3p expression was associated with poor progression-free (p = 0.031) and overall (p = 0.026) survival in HGSC patients. Interacting miRNA and mRNA targets, including those of a TP53-related pathway presented previously, were identified in HGSC.

**Conclusions:**

Several miRNAs differentially expressed between HGSC, CCC and OSE have been identified, suggesting a carcinogenetic role for these miRNAs. miR-200 family members, targeting EMT drivers, were mostly overexpressed in both subgroups, among which miR-200c-3p was associated with survival in HGSC patients. A set of miRNAs differentiates CCC from HGSC, of which miR-509-3-5p and miR-509-5p are the strongest classifiers. Several interactions between miRNAs and mRNAs in HGSC were mapped.

## Background

Ovarian cancer is the fourth and fifth most frequent cause of cancer death in women in Norway and the U.S., respectively [1,2]. Two-thirds of patients have advanced-stage disease (International Federation of Gynecology and Obstetrics [FIGO] stage III-IV) at diagnosis, resulting in 5-year survival at <30% [1,2].

Ovarian carcinoma (OC) constitutes about 90% of ovarian cancers, and is a heterogeneous group of tumors, encompassing several distinct subgroups with respect to molecular profiles, biological behavior and clinical features [3-6]. Nevertheless, OC patients generally receive similar, non-individualized treatment. Therefore, improved insight into the molecular characteristics of the different OC subgroups may aid in development of a more subgroup-specific treatment, thereby improving prognosis.

microRNAs (miRNAs) are short, non-coding RNA molecules, which by targeting mRNAs cause mRNA degradation or translational repression [7]. Since a single miRNA may have multiple different mRNA targets and conversely, a given mRNA might be targeted by multiple miRNAs, miRNAs play a central role in regulating gene expression. Alterations in miRNA expression level may consequently alter the level of a wide spectrum of mRNAs and subsequently cellular functions.

miRNAs show abnormal expression patterns in different cancer forms [8]. Some act as tumor suppressor genes or oncogenes and may therefore be important in cancer development [9,10]. Various gene expression analysis approaches, including microarrays, have identified aberrantly expressed miRNAs in OC [9,11-26], of which some are related to progression [13], outcome [18-24] and chemotherapy resistance [23-25]. However, the studies have in general utilized non-subgroup specific tumors [11], and only a few included normal ovarian surface epithelium (OSE) [15-17], which has been shown to be valid control material [27,28].

The aim of this study was to identify miRNAs differentially expressed between moderately and poorly differentiated serous OC, referred to as high-grade serous OC (HGSC), clear cell OC (CCC) and scrapings from OSE, and to evaluate their association with clinical parameters, including survival. To identify potential key molecular pathways of the carcinogenesis of HGSC, differentially expressed miRNAs and mRNAs identified previously [29] were linked. We have identified several miRNAs differentially expressed between HGSC, CCC and OSE, including miR-200c-3p with apparent clinical relevance in HGSC. Several interactions of potential oncogenic function between aberrantly expressed miRNAs and mRNAs in HGSC have also been mapped, including interactions between miR-200 members and the epithelial-mesenchymal transition (EMT) regulators ZEB1 and ZEB2.

## Methods

### Ethics statement

The study was approved by the Regional Committee of Medical and Health Research Ethics of South-Eastern Norway (ref.no.530-02163 and S-04300) and all participants signed informed consent.

### Patients and material

Women were enrolled prior to operations for gynecological diseases at Oslo University Hospital (OUH) during 2003–2012. Patient information was obtained from hospital records and preoperative interviews. Patients were evaluated routinely [29] and follow-up data, including clinical examinations, laboratory analyses and imaging were available for all patients. CA125 level was used as marker for therapy response. CA125 normalization (<35 kU/L) was defined as optimal when achieved within four cycles of chemotherapy. Time until progression and time until death were defined as the time interval from the date of surgery to the date of first confirmed disease recurrence and to the date of death, respectively. Disease progression was based on CA125 level increase according to GCIG (Gynecologic Cancer Intergroup) criteria (http://www.gcig.igcs.org) and verified clinical relapse, and the date of first event was used. Clinical data were current as of March 20, 2013.

Tumors comprised primary OC obtained pre-chemotherapy. OSE samples were collected from patients with benign diseases, as previously described [27]. Tumors were snap-frozen in liquid nitrogen immediately after harvesting, whereas OSE samples were transferred to QiaZol solution (Invitrogen, Carlsbad, CA). All samples were stored at -80°C until processed.

The histological classification and clinical staging were according to the World Health Organization classification and FIGO, respectively. Tumors were reviewed by a gynecological pathologist (BD) to confirm the histological type and grade. A frozen section from all biopsies was examined prior to RNA isolation to ensure a tumor component of at least 50% and absence of necrosis.

### RNA preparation

Frozen tumors (<50 mg) were homogenized directly for 3 minutes in 700 μl QIAzol using a TissueLyzer (Qiagen, Hilden, Germany). Total RNA was extracted using the miRNeasy Mini Kit (Qiagen) and Phase Lock Gel™ Heavy (5 PRIME GmbH, Hamburg, Germany). RNA was quantified with a NanoDrop^®^ ND-1000 Spectrophotometer (Saveen Werner, Malmö, Sweden), and quality assessed on Agilent 2100 Bioanalyzer RNA 6000 Nano Kits (Agilent Technologies, Palo Alto, CA). All samples showed adequate RNA quantity and quality.

### Global miRNA expression profiling

Global miRNA expression was analyzed in 12 HGSC, 9 CCC and 9 OSE samples. Total RNA (400 ng) was used for biotin labeling of miRNA by the Genisphere FlashTag HSR kit following the manufacturer’s recommendations (Genisphere, Hatfield, PA). Labeled miRNAs were hybridized to the GeneChip miRNA 2.0 Array (Affymetrix, Santa Clara, CA), representing 1,105 mature human miRNAs, as recommended by the manufacturer. Arrays were washed and stained using the FS-450 fluidics station (Affymetrix). Signal intensities were detected by Hewlett Packard Gene Array Scanner 3000 7G (Hewlett Packard, Palo Alto, CA). Microarray data were deposited in NCBI’s Gene Expression Omnibus (GEO) [30] and are accessible through GEO Series accession number GSE47841 (http://www.ncbi.nlm.nih.gov/geo/query/acc.cgi?acc=GSE47841).

### Quantitative reverse transcription-polymerase chain reaction (RT-qPCR)

Selected candidate miRNAs were validated by RT-qPCR in all samples analyzed by global miRNA expression profiling (except one excluded) and in additional samples, totaling 35 HGSC, 19 CCC and 9 OSE samples. Custom-made TaqMan^®^ Low Density Array (TLDA) cards for human miRNA expression analysis (Applied Biosystems, Life Technologies, Carlsbad, CA) were used for quantification of specific miRNAs, each card allowing 384 simultaneous qPCR reactions of 24 different miRNAs run in duplicates. Included were two selected reference genes and one mandatory control (U6 (mammu6) snRNA).

Total RNA (350 ng) was applied for reverse transcription (RT) with stem-looped RT primer-mix, enabling synthesis of cDNA from mature miRNAs. Unbiased custom-based pre amplification was performed according to protocols, using gene-specific forward and reverse primers. The PCR reactions were performed on Unocycler (VWR International, B-3001 Leuven, Belgium). The TLDA cards were used for further PCR-amplification on a ViiA7™ Real Time PCR system thermocycler and analyzed with ViiA7 RUO Software (Applied Biosystems, Life Technologies).

Relative gene expression levels were calculated using the comparative crossing threshold method of relative quantification (∆∆Cq method) [31,32], and presented as relative quantification cycle (ΔCq) and fold change (FC) values. ΔCq was designated as the mean Cq (mean of duplicates) of a miRNA in a sample subtracted by the mean Cq (mean of duplicates) of two reference genes in the same sample. Based on recommendations from the manufacturer and comparison between the microarray and RT-qPCR analyses, Cq expression cutoff was set to 30, which was applied for calculations. For analyzing associations with clinical parameters, ΔΔCq was calculated as mean ΔCq of the OSE controls subtracted by ΔCq of each tumor sample. For comparison of mean expression levels between different groups, ΔΔCq was calculated as mean ΔCq of one group subtracted by mean ΔCq of another group (∆Cq_OSE_ - ∆Cq_HGSC;_ ∆Cq_OSE_ - ∆Cq_CCC;_ ∆Cq_HGSC_ - ∆Cq_CCC_). FC was designated as 2^ΔΔCq^.

All miRNAs analyzed were from Homo sapiens (hsa) and the prefix hsa was therefore excluded.

### Ingenuity pathway analysis (IPA)

Data were analyzed through the use of IPA (Ingenuity^®^ Systems, http://www.ingenuity.com), which was used for identifying biological functions and related diseases and for mapping mRNA-miRNA interactions.

### Statistical analysis

For computational analysis of the microarray data, scanned images were processed using the AGCC (Affymetrix GeneChip Command Console) software, and the CEL files were imported into Partek Genomics Suite software (PGS; Partek, Inc., St Louis, MO). The Robust Multichip Analysis (RMA) algorithm was applied for generation of relative signal values and normalization. For expression comparisons of different groups, a 1-way ANOVA model followed by calculation of FDR was used. Results were expressed as FC and p-values. Signal values were subjected to a non-supervised cluster analysis using the Euclidean/average linkage algorithm.

Associations between signal values and progression-free survival (PFS) and overall survival (OS) were evaluated by Cox regression analyses followed by FDR correction. FDR q-values of 0.1 and 0.25 were used as significance levels for PFS and OS, respectively.

When comparing ΔCq values in different histological subgroups, a two-sided independent samples t-test was used since the ΔCq values were close-to-normally distributed. Associations between FC values of the RT-qPCR analyses and clinical parameters were evaluated. In order to decide whether expression of a miRNA was significantly associated with PFS and OS, Cox regression analyses were used. When significant, Kaplan-Meier plots were used to estimate survival curves for tertiles of the expression variable. To compare miRNA expression levels in two groups of patients, a two-sided Mann–Whitney U-test was used, since the FC expression levels were not normally distributed. The results for each group are presented as medians.

A significance level of 1% was used for differential miRNA expression, and 5% when analyzing associations with clinical parameters. Statistical analyses were performed using the SPSS-PC package (Version 20, Chicago IL).

## Results

### Patient characteristics

Clinicopathologic data for the RT-qPCR cohort are shown in Table 1. All HGSC patients were diagnosed with FIGO stage IIIc/IV, whereas CCC patients were diagnosed at all stages due to limited patient material. The patients had no disease other than OC influencing survival, were Caucasian, and except for 1 with HGSC and 2 with CCC all were in good preoperative condition [27].

**Table 1:** Clinicopathological and laboratory information for patients selected for RT-qPCR analysis

**Parameter**		**HGSC**^***a***^**, n = 35**	**CCC**^***a***^**, n = 19**
Age; mean ± SD (range)		64.0 ± 11.3 (45–87)	63.9 ± 15.3 (28–83)
Preoperative CA125 (kU/L); mean ± SD		3023 ± 4129	1438 ± 2198
FIGO stage	I	0	10
	II	0	3
	III	25	5
	IV	10	1
Residual disease	0 cm	3	13
	<2 cm	9	4
	>2 cm	23	2
Start of chemotherapy (days after surgery); mean ± SD		27.7 ± 11.6	25.7 ± 13.8
CA125 response^*b*^	Yes	31	15
	No	1	0
Optimal CA125 normalization^*b*^	Yes	20	13
	No	14	3
Median time (months) until progression (95% CI)		10 (7–13)	NA^*c*^
Median time (months) until death (95% CI)		26 (18–34)	105 (35–175)
Status at last follow-up^*d*^	NED	1	11
	AWD	1	1
	DOD	33	6
	DOUC	0	1

^*a*^HGSC: High-grade serous ovarian carcinoma; CCC: Clear cell ovarian carcinoma.

^*b*^According to the GCIG criteria (http://www.gcig.igcs.org). For HGSC: Two patients who received no postoperative treatment due to poor general condition, one patient with preoperative CA125 < 70; For CCC: One patient who received no postoperative treatment due to poor condition, three patients with preoperative CA125 < 70.

^*c*^Could not be calculated since the Kaplan-Meier survival curve stays above 50%.

^*d*^NED = No evidence of disease; AWD = Alive with disease; DOD = Dead of disease; DOUC = Dead of unrelated cause.

Primary surgery was performed in all patients. With the exception of 4 HGSC and 5 CCC patients, all received platinum-based chemotherapy. The 4 HGSC patients were considered to be in too poor general condition to tolerate chemotherapy. Among CCC patients, 1 received paclitaxel-based treatment, 1 was in too poor general condition, and 3 did not receive chemotherapy due to FIGO stage IA.

### Global miRNA expression analyses

**Figure 1:**
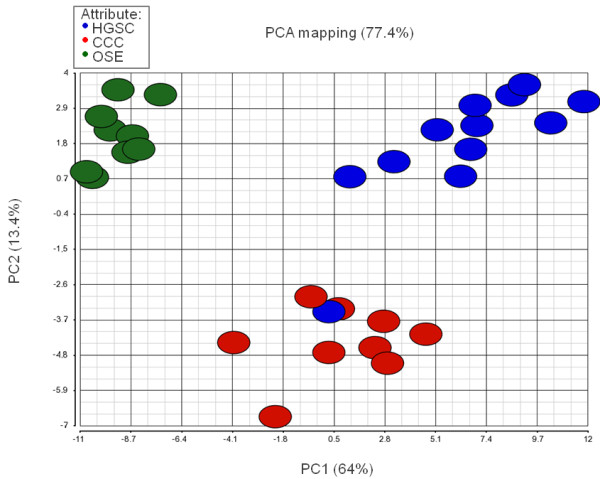
**Principal component analysis (PCA).** A two-dimensional PCA of 78 miRNAs found to be differentially expressed following ANOVA and application of FDR < 0.01% based on global miRNA expression analyses in 12 high-grade serous ovarian carcinomas (HGSC; blue), 9 clear cell ovarian carcinomas (CCC; red) and 9 ovarian surface epithelium (OSE; green).

**Figure 2:**
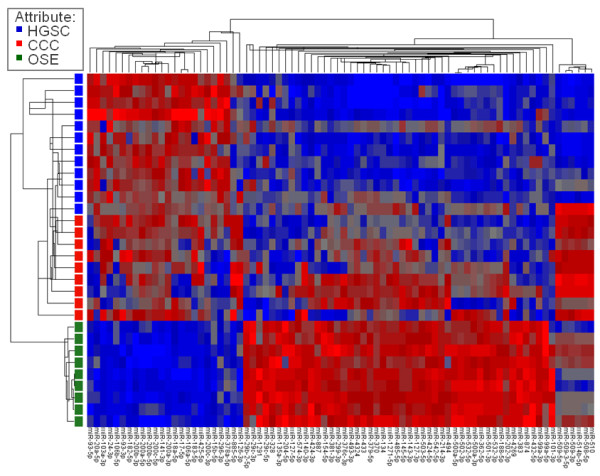
**Cluster analysis heatmap.** Cluster analysis heatmap of expression levels (signal values) of 78 miRNAs found to be differentially expressed following ANOVA and application of FDR < 0.01% based on global miRNA expression analyses in 12 high-grade serous ovarian carcinomas (HGSC; blue), 9 clear cell ovarian carcinomas (CCC; red) and 9 ovarian surface epithelium (OSE; green). Each column represents a miRNA and each row a sample. The more over- and under-expressed the miRNA, the brighter the red and blue color, respectively.

Seventy-eight miRNAs were differentially expressed between HGSC, CCC and OSE applying a FDR <0.01%. Complete p- and FC values are available in Additional file 1. Principal component analysis showed that these miRNAs could distinguish the 3 groups almost perfectly (Figure 1). Cluster analysis, visualized by a heatmap (Figure 2) showed almost perfect segregation of the 3 groups. Striking differences were observed between HGSC and OSE samples, whereas CCC had an intermediate profile. Moreover, miR-508-5p, miR-509-3p, miR-509-5p, miR-509-3-5p, miR-510 and miR-514b-5p clearly distinguished HGSC from CCC. OSE control samples were homogeneous.

### Evaluation of associations between global miRNA expression and survival

Associations between miRNAs with signal values >7 (n = 297) and PFS (FDR q < 0.1) and OS (FDR q < 0.25) were separately evaluated in HGSC and CCC. No statistically significant associations were found. However, when not corrected for multiple testing, 11 miRNAs had p < 0.05, indicating an association with survival. Of these, miR-505-5p, miR-1281 and miR-29b-2-5p had the lowest p-values (p < 0.03), all with potential association with survival in HGSC. These miRNAs were among the miRNAs chosen for RT-qPCR validation and subsequent evaluation for association with outcome in the extended patient cohort. Noteworthy, only miR-29b-2-5p was among the differentially expressed miRNAs shown in Figure 2.

### RT-qPCR validation of selected miRNAs

Twenty-one miRNAs and 2 reference genes were selected for RT-qPCR validation in the extended patient material. Of these, 18 miRNAs (Table 2) were predominantly selected based on differential expression (Figure 2). All miRNAs with FC > ±20 (n = 16) and 2 of the mRNAs with FC > ±15 were included, reaching a highest FC value of 105. Additionally, the 3 above-mentioned miRNAs were selected based on possible association with survival. miR-24 and miR-26a were selected as reference genes, having the lowest expression variation (0.11 and 0.10, respectively) in the global miRNA analysis. Their mean value reduced the variation to 0.029, and their mean Cq value was therefore used for calculations.

**Table 2:** Differentially expressed miRNAs between HGSC, CCC and OSE of global miRNA expression profiling* selected for RT-qPCR validation

	**HGSC vs. OSE**^*a*^		**CCC vs. OSE**^*a*^		**CCC vs. HGSC**^*a*^	
**miRNAs**	**p-values**	**FC values**	**p-value**	**FC value**	**p-value**	**FC value**
**miR-134**	8.3 × 10^-8^	-16.7^*b*^			1.0 × 10^-4^	5.8
**miR-141-3p**	1.1 × 10^-11^	46.1	2.4 × 10^-10^	34.9		
**miR-182-5p**	6.0 × 10^-9^	30.2	1.4 × 10^-8^	32.7		
**miR-200a-3p**	7.3 × 10^-10^	33.6	1.3 × 10^-9^	38.8		
**miR-200a-5p**	3.0 × 10^-13^	33.5	6.9 × 10^-12^	26.5		
**miR-200b-3p**	1.1 × 10^-9^	29.1	2.9 × 10^-8^	21.1		
**miR-200c-3p**	1.2 × 10^-12^	16.5	1.2 × 10^-11^	15.0		
**miR-202-3p**	8.0 × 10^-6^	-36.9			2.3 × 10^-4^	16.3
**miR-205-5p**	4.9 × 10^-5^	105.1			3.1 × 10^-3^	-23.1
**miR-383**	8.2 × 10^-12^	-33.7	1.5 × 10^-11^	-38.7		
**miR-424-5p**	2.6 × 10^-9^	-26.0	4.0 × 10^-6^	-10.1		
**miR-508-5p**			4.4 × 10^-3^	11.6	3.1 × 10^-6^	75.0
**miR-509-3p**	4.3 × 10^-3^	-10.3			2.6 × 10^-6^	83.4
**miR-509-5p**			5.6 × 10^-4^	11.4	1.8 × 10^-6^	34.0
**miR-509-3-5p**	3.9 × 10^-3^	-10.2			1.9 × 10^-6^	84.6
**miR-510**	9.3 × 10^-3^	-5.2	7.9 × 10^-3^	6.1	3.0 × 10^-6^	31.7
**miR-513a-5p**			4.8 × 10^-3^	7.4	4.1 × 10^-6^	33.5
**miR-514b-5p**	9.7 × 10^-3^	-6.5	3.8 × 10^-3^	9.8	1.3 × 10^-6^	63.6

*Affymetrix GeneChip miRNA 2.0 Arrays. ^*a*^HGSC: High-grade serous ovarian carcinoma. OSE: ovarian surface epithelium. CCC: Clear cell ovarian carcinoma. ^*b*^‘-’ illustrates underexpression. FC: Fold change. P-values are calculated on original data (before FDR corrections).

All miRNAs selected based on differential expression were verified as markedly differentially expressed, with p-values varying from 10^-7^ to 10^-21^ and FC values up to 95 (Table 3). When comparing HGSC with OSE, 7 and 6 miRNAs were over- and under-expressed in HGSC, respectively. According to FC values, miR-205-5p was the most overexpressed (FC = 74), followed by miR-200c-3p, miR-182-5p, miR-141-3p and miR-200b-3p. When comparing CCC with OSE, 11 and 2 miRNAs were over- and underexpressed, respectively, including 8 common with the HGSC vs. OSE analysis. miR-182-5p best distinguished CCC from OSE (FC = 66), followed by miR-200a-3p, miR-200c-3p, miR-200a-5p and 200b-3p. All these miRNAs were overexpressed, whereas miR-383 was the most underexpressed in both HGSC and CCC.

**Table 3:** Differentially expressed miRNAs (p < 0.01) between HGSC, CCC and OSE verified by RT-qPCR

	**HGSC vs. OSE**^*a*^		**CCC vs. OSE**^*a*^		**CCC vs. HGSC**^*a*^	
**miRNAs**	**p-values**	**FC values**	**p-values**	**FC values**	**p-values**	**FC values**
**miR-134**	8.7 × 10^-11^	-5.7^*b*^			3.1 × 10^-6^	4.3
**miR-141-3p**	1.7 × 10^-18^	40.3	7.2 × 10^-11^	45.3		
**miR-182-5p**	9.5 × 10^-15^	42.4	1.2 × 10^-8^	66.2		
**miR-200a-3p**	3.6 × 10^-5^	33.0	9.3 × 10^-10^	57.8		
**miR-200a-5p**	3.1 × 10^-15^	33.8	4.3 × 10^-11^	53.0		
**miR-200b-3p**	5.3 × 10^-18^	38.8	3.7 × 10^-11^	51.0		
**miR-200c-3p**	6.0 × 10^-21^	48.2	3.2 × 10^-12^	53.4		
**miR-202-3p**	1.3 × 10^-14^	-14.7			1.6 × 10^-7^	10.1
**miR-205-5p**	9.0 × 10^-9^	74.3			4.4 × 10^-3^	-8.4
**miR-383**	2.2 × 10^-14^	-36.6	9.8 × 10^-10^	-15.1	2.2 × 10^-3^	2.4
**miR-424-5p**	3.1 × 10^-13^	-10.7	3.5 × 10^-4^	-4.2	1.6 × 10^-3^	2.5
**miR-508-5p**			3.5 × 10^-3^	10.1	1.0 × 10^-8^	27.5
**miR-509-3p**					2.0 × 10^-7^	46.3
**miR-509-5p**	5.0 × 10^-3^	-4.1	2.4 × 10^-3^	13.3	1.3 × 10^-8^	54.7
**miR-509-3-5p**	1.1 × 10^-4^	-11.0			2.2 × 10^-8^	95.3
**miR-510**			2.5 × 10^-3^	9.0	8.7 × 10^-10^	32.9
**miR-513a-5p**			6.6 × 10^-4^	6.2	9.1 × 10^-7^	8.3
**miR-514b-5p**			9.7 × 10^-5^	12.1	2.3 × 10^-9^	25.8

^*a*^HGSC: High-grade serous ovarian carcinoma. OSE: ovarian surface epithelium. CCC: Clear cell ovarian carcinoma. ^*b*^‘-’ illustrates underexpression. FC: Fold change.

Twelve miRNAs distinguished CCC from HGSC, all except 1 being overexpressed in CCC. The miRNA with highest FC values was miR-509-3-5p (FC = 95), followed by miR-509-5p, miR-509-3p, miR-510 and miR-508-5p.

Experimental information annotated from IPA for these specific miRNAs is provided in Table 4. As shown, these miRNAs are active regulators of the expression of several cancer-related mRNAs, including ZEB1, ZEB2, VIM, VEGFA, NTRK3 and SPDEF, and most of the miRNAs are cancer-related. The table also highlights differentially expressed miRNAs (Tables 2 and 3) and mRNAs (FC > ±1.5) identified previously [29] in HGSC vs. OSE, among others ZEB1, ZEB2 and VIM, interacting inversely with miR-200c-3p, miR-200a-3p, miR-205-5p and miR-141-3p. Complete HGSC vs. OSE FC values for the mRNAs listed in Table 4 are provided in Additional file 2.

**Table 4:** IPA based experimentally observed information for differentially expressed miRNAs and differential expression (FC > ±1.5) of their regulated mRNA targets in HGSC

**miRNAs**	**mRNA targets**	**Cancer association**	**OC association**
**miR-134↓**		x	
**miR-141-3p↑**	TGFB2**↓**, ZEB2**↓**, JAG1, BAP1, CLOCK, ELMO2, ERBB2IP, KLHL20, MAP2K4, PLCG1, PTPRD, WDR37	x	x (EC)
**miR-182-5p↑**	FOXO3, ADCY6, CASP2**↑**, CLDN17, NCAM1**↓**, NFASC**↓**, RARG, BCL2L14**↑**, CARD11**↑**, CASP10**↑**, CASP12, CDH1**↑**, CDH4, CDK6, CLDN15**↓**, COL11A2, COL4A4**↓**, FNDC3A**↓**, FOXO1**↓**, GADD45G**↓**, GJA3, IGF1R**↓**, INHBC, ITGA4, LRP6, MALAT1**↓**, MITF**↓**, MTSS1, NLGN2, PGF, PIK3CA**↑**, RPS6KB1, SOS1, VWF**↑**	x	x (EC)
**miR-200a-3p↑**	CTNNB1, VIM**↓**, ZEB1**↓**, ZEB2**↓**, BAP1, CDK6, CDKN1B**↓**, CTBP2, CYP1B1↑, ELMO2, ERBB2IP, KLHL20, PLCG1, PTPRD**↓**, TUBB**↑**, WDR37, ZFPM2**↓**	x	x (EC, ROC)
**miR-200a-5p↑**		x	
**miR-200b-3p↑**	VIM**↓**, ZEB1**↓**, ZEB2**↓**, BAP1, ELMO2, ERBB2IP, ERRFI1, KLHL20, PLCG1, PTPRD**↓**, RERE, WASF3, WDR37, ZFPM2**↓**	x	x (ROC)
**miR-200c-3p↑**	CDH1**↑**, PTPN13**↓**, ZEB1**↓**, ZEB2**↓**, FHOD1, PPM1F, JAG1, MARCKS, VIM**↓**, CDKN1B**↓**, ERRFI1**↓**, PLCG1	x	x (EC)
**miR-202-3p↓**			
**miR-205-5p↑**	ERBB3**↑**, F Actin, INPPL1**↑**, MED1, VEGFA**↑**, ZEB1**↓**, ZEB2**↓**, PRKCE	x	x (EC)
**miR-383↓**			
**miR-424-5p↓**	FGFR1, MAP2K1, NFIA**↓**, PLAG1	x	
**miR-508-5p**			
**miR-509-3p↓**	NTRK3		
**miR-509-5p↓**			
**miR-509-3-5p↓**			
**miR-510↓**	HTR3E, SPDEF**↑**	x	
**miR-513a-5p**	CD274	x	
**miR-514b-5p↓**			

IPA: Ingenuity Pathway Analysis. HGSC: High-grade serous ovarian carcinoma. OC: Ovarian carcinoma. EC: Endometrioid OC. ROC: Recurrent OC. Over- and underexpressed miRNAs (based on Tables 2 and 3) and mRNAs (based on global gene expression analysis [29]) in HGSC vs. ovarian surface epithelium (OSE) are indicated by upward and downward arrows, respectively. A complete list of HGSC vs. OSE FC values for the mRNA targets is available in Additional file 2.

### Associations between validated miRNA expression and clinical parameters

**Figure 3:**
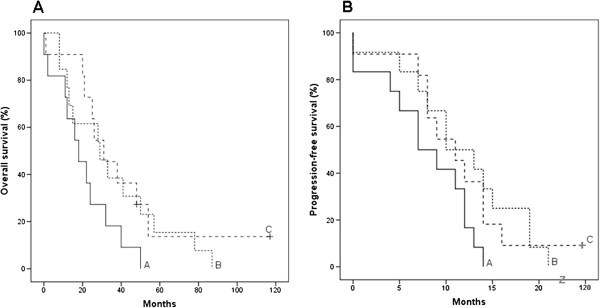
**Kaplan-Meier survival curves for miR-200c-3p expression in HGSC patients.** Overall survival (OS) curves **(A)** and progression-free survival (PFS) curves **(B)** according to miR-200c-3p expression level (FC) tertiles in patients with high-grade serous ovarian carcinomas (HGSC, n = 35) based on significant association between miR-200c-3p and PFS (p = 0.031) and OS (p = 0.026). A: High expression. B: Intermediate expression. C: Low expression. The symbol “+” indicates censoring. Median time until progression and death is given in Table 1.

All miRNAs validated by RT-qPCR were evaluated for association with PFS, OS, optimal CA125 normalization and residual disease (RD) in patients included in the RT-qPCR analyses. In HGSC, miR-200c-3p was found to be associated with PFS (p = 0.031) and OS (p = 0.026). The miR-200c-3p FC expression level was divided into tertiles, and Kaplan-Meier plots made (Figure 3). Patients with highest tertile level had shorter OS than patients with intermediate or lowest levels, with median time until death of 18 and 30 months, respectively (Figure 3A). Patients with the highest tertile level had shorter PFS compared with patients with lowest levels, with median time until progression of 7 and 11 months, respectively (Figure 3B). No association was found between the miRNAs and CA 125 normalization or RD (cut-off at 2 cm) in HGSC. The 3 miRNAs selected for RT-qPCR based on possible association with survival were not found to be associated with outcome.

In CCC, no associations with PFS or OS were found. However, patients with macroscopic RD (cut-off at 0 cm) had significantly lower miR-202-3p (p = 0.018) and miR-1281 (p = 0.035) levels (n = 6; median FC = -5.3 and -2.0, respectively) than patients without RD (n = 13; median FC = 1.6 and -1.2, respectively). Associations with CA 125 normalization could not be evaluated in CCC, since all but 3 patients achieved optimal CA 125 normalization.

### Ingenuity pathway analysis (IPA)

To identify miRNA-mRNA interactions in HGSC, differentially expressed miRNAs in HGSC vs. OSE were linked to differentially expressed mRNAs in HGSC vs. OSE identified previously [29]. miRNAs and mRNAs of the microarray analyses (ANOVA, FDR < 5%) were imported to the IPA software and filtered for interactions. When including miRNAs and mRNAs with FC ≥ ±10, interactions of inverse miRNA-mRNA expression pairing (55.4% of the interactions), interactions experimentally observed and of high predicted confidence, 19 miRNAs targeting 47 mRNAs (Table 5) were found. All but 3 miRNAs are included in Figure 2. Core analysis was performed, and selected cancer-related functions are shown in Table 5. Fifty-four RNAs were cancer-related, of which 11 mRNAs and 8 miRNAs were OC-related (italics). Thirty-one and 10 molecules were related to cell proliferation and cell cycle, respectively. For a detailed evaluation of the quality of the predicted miRNA-mRNA interactions of Table 5, a plot showing the Context + score as well as number of conserved binding sites of these interactions (TargetScan) is given in Additional file 3.

**Table 5:** Interacting miRNAs and mRNAs in HGSC - differentially* and inversely expressed

**miRNAs and mRNA targets**	**FC values**
miR-134^1,5^	-16.7
KLHL14^1^, *PAX8*^1,3,5^	9.6 – 29.3
*miR-141-3p*^1,2,6^/ *miR-200a-3p*^1,2,6^	46.1
*FOXP2*^1,2,5^, HLF^1^, PCDH9^5^, *PEG3*^1,6^, SCN7A^1^, SDC2^1,2,3,4,5,6^	-9.6 – -24.7
*miR-182-5p*^1,2^	30.2
ANGPTL1^1,2,5^, CACNB2^1,5^, *FOXP2*^1,2,5^, KCNMB2^1,5^, PID1^1^, SDC2^1,2,3,4,5,6^, TMEM150C	-9.6 – -14.6
*miR-183-5p*^1,2,6^	11.7
*ABCA8*^1^, HLF^1^	-11.6 – -17.8
miR-187-3p^1^	12.8
TSPAN5^1,2^	-11.3
*miR-200b-3p*^1,2,6^/ *miR-200c-3p*^1,2,6^	29.1
CACNB2^1,5^,CDH11^1,2,5,6^, COL4A3^1,2,5,6^, GPM6A^1^, HLF^1^, HS3ST3A1^1^, LEPR^1,2,4,5,6^, MCC^1,2^, NEGR1^1,2,6^, SDC2^1,2,3,4,5,6^	-9.6 – -21.6
miR-202-3p	-36.9
*RRM2*^1,2,4,6^	16.9
*miR-203-3p*^1,2,6^	13.6
ANGPTL1^1,2,5^, EDNRA^1,2,6^, *FOXP2*^1,2,5^,GNG4^2,6^, IGFBP5^1,2,3,4,5,6^, NEGR1^1,2,6^, SMAD9^1^	-9.7 – -15.0
*miR-205-5p*^1,6^	105.1
BAMBI^1,2,5,6^, NR3C2^1,2,5,6^, *PEG3*^1,6^	-10.1 – -24.7
miR-376c-3p^1^	-11.7
EHF^1,2,3,6^, *LRP8*^1,5,6^	11.9 – 12.6
miR-379-5p	-10.3
KLHL14^1^	29.3
miR-381-3p^1^	-12.6
EGFL6^1^, *NOTCH3*^1,2,3,5,6^, *RRM2*^1,2,4,6^	9.7 – 16.9
miR-383	-33.7
MAL2	32.8
*miR-424-5p*^1,2,3,4,5,6^	-26.0
AHNAK2^1^, *CCNE1*^1,2,3,4,6^, *ESRP1*^1,6^, *HMGA1*^1,2,3,4,6^, LAMP3^1,2^, *PSAT1*^1^, *UCP2*^2,5^, VAMP8^1,2^	9.9 – 24.0
miR-485-5p^1^	-13.6
KRT7^1,3,4^, *LRP8*^1,5,6^, ST14^1^	10.5 – 16.2
miR-887	-9.6
TMEM139	11.7
miR-4324	-12.5
*ERBB3*^1,2,3,5,6^, GALNT6	17.9 – 23.7

HGSC: High-grade serous ovarian carcinoma. *ANOVA, FDR < 5%, FC ≥ ±10 in HGSC vs. ovarian surface epithelium. All interactions are of high predicted confidence, whereas the interactions between *miR-424-5p* and *CCNE1, HMGA1, PSAT1 and UCP2 are* experimentally observed. ^1, 2, 3, 4, 5, 6^Related to cancer (italicized for ovarian cancer), cellular growth and proliferation, cell cycle, DNA replication, recombination and repair, cell-to-cell signaling and interaction, cellular development, respectively. Results were generated through the use of Ingenuity Pathway Analysis.

**Figure 4:**
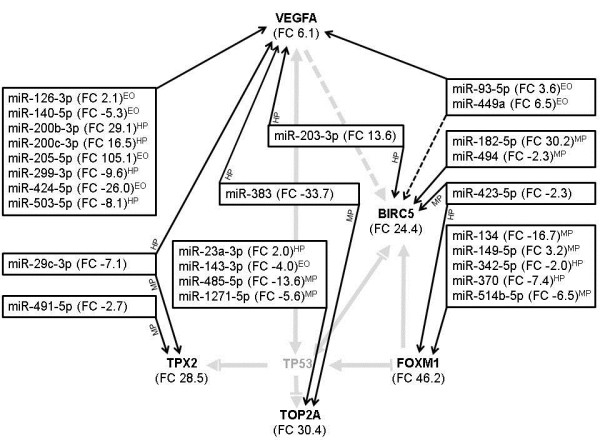
**Differentially expressed (FC ≥ 2) miRNAs in HGSC targeting a HGSC molecular pathway of differentially expressed mRNAs **[29]**.** Calculated differential expression is between high-grade serous ovarian carcinomas (HGSC) and ovarian surface epithelium, for miRNAs based on global gene expression analysis (ANOVA, FDR < 5) and for mRNAs based on RT-qPCR analyses [29]. **→** acts on (— direct interaction, -- indirect interaction), ⟂ inhibits. FC: Fold change. EO: Experimentally observed interactions. HP: Interactions of high predicted confidence. MP: Interactions of moderate predicted confidence. The abbreviations of the miRNA-mRNA interactions are generally placed after the miRNA FC values, but are placed on the arrow of the miRNA-mRNA interaction for miRNAs having several targets with different interaction type. Results were generated through Ingenuity Pathway Analysis.

We previously presented a HGSC pathway comprising VEGFA, FOXM1, TPX2, BIRC5 and TOP2A, all significantly overexpressed in HGSC vs. OSE and directly interacting with TP53 [29]. Through IPA, these mRNAs were linked to differentially expressed miRNAs in HGSC vs. OSE of the microarray analysis (ANOVA, FDR < 5%, FC > ±2). When inverse and similar miRNA-mRNA expression pairing and all confidence levels were included, 26 miRNAs and 30 interactions were found (Figure 4). Of these, 7 and 12 were experimentally observed and of high predicted confidence, respectively. Among the miRNAs, 16 were under- and 10 overexpressed. All but 9 miRNAs are included in Figure 2.

## Discussion

In this study, a number of miRNAs distinguishing HGSC and CCC from OSE, as well as CCC from HGSC have been identified, including a set validated by RT-qPCR. These miRNAs could be involved in the biology of these OC subgroups.

The most differentially expressed miRNAs in both HGSC and CCC compared with OSE were miR-200 family members, including miR-200a-3p, miR-200b-3p, miR-200c-3p and miR-141-3p. These miRNAs are aberrantly expressed in different cancers [33-36], and have been found to be overexpressed in serous and clear cell OC, although few CCC were analyzed [17,22,26].

miR-200 family members have been demonstrated to regulate EMT by targeting ZEB1 and ZEB2, resulting in altered expression of the cell-cell adhesion molecule E-cadherin [37-40]. E-cadherin down-regulation is apparently important in cancer progression, facilitating cell detachment and metastasis. At a favorable distant location, cells may undergo mesenchymal-epithelial transition (MET) and re-express E-cadherin. This is supported by the finding of elevated E-cadherin and reduced ZEB1 in metastatic epithelial ovarian cancer [41], as well as by our findings of overexpressed miR-200 family members and underexpression of ZEB1 and ZEB2 in metastatic HGSC. ZEB1 and ZEB2 are also targets of miR-205-5p [37], which was highly overexpressed in HGSC compared with OSE and CCC.

miR-200c-3p and miR-200b-3p, having similar seed sequences, have been shown to decrease VIM expression and thereby its protein vimentin [39]. Vimentin is found in various non-epithelial cells, especially mesenchymal cells, and is used as marker for EMT during metastasis. Elevated expression of miR-200c-3p and miR-200b-3p, resulting in reduced vimentin levels, is therefore expected in metastatic cancer, where epithelial features are important for re-colonization, in concordance with our findings.

Interestingly, among the IPA documented mRNA targets of the differentially expressed miRNAs in this study, ZEB1 and ZEB2 were among the most underexpressed mRNAs in HGSC compared with OSE. Even though the miRNA-mRNA interactions are not verified in the HGSC material presented, the inverse expression of miR-200 members and ZEB1, ZEB2 and VIM, as well as of miR-205-5p and ZEB1 and ZEB2, support a probable interaction also in HGSC.

miR-182-5p had the highest FC in CCC compared with OSE. This miRNA regulates the expression of PIK3CA, a frequently mutated gene in CCC and a candidate for targeted therapy [42]. Little is known about miR-200a-5p, although it has been related to colorectal cancer [36].

To the best of our knowledge, the present study is the first to identify differentially expressed miRNAs in a relatively large CCC series. The miRNAs most clearly separating CCC from HGSC were miR-509-3-5p and miR-509-5p, having similar seed sequences, as well as miR-509-3p and miR-510. miR-509-3p has been shown to target NTRK3 [43], encoding the receptor tyrosine kinase TrkC, which is involved in the oncogenic PIK3CA pathway. miR-509-3p, miR-509-3-5p and also miR-513a-5p have been found overexpressed in stage I OC [23], and miR-509-5p have been found to inhibit cancer cell proliferation [44]. miR-510 targets SPDEF [45], which have been found underexpressed in OC compared with breast carcinoma [46]. Our findings of an underexpressed miR-510 and overexpression of SPDEF in HGSC support an interaction also in this cancer subgroup.

In spite of the relatively small sample size, high level of miR-200c-3p was found to be associated with short PFS and OS in HGSC, indicating it may be a prognostic marker for HGSC. This finding is in accordance with a study analyzing miRNA expression in SC vs. normal ovaries [22]. The fact that most of its differentially expressed and experimentally observed mRNA targets were found underexpressed may bolster the conclusion that miR-200c-3p is associated with survival. This miRNA has also been associated with survival in stage I OC patients [47] and chemotherapy response [48]. miR-200c-3p was among the most differentially expressed miRNAs in both HGSC and CCC compared with OSE separately, and had the lowest p-value in both comparisons. miR-200c-3p has previously been found to be overexpressed in SC [22,26], HGSC cell lines [49], serum from HGSC patients [49] and in a small series of CCC [26]. Based on the relatively small number of HGSC patients, the findings of the survival analysis should be verified in an extended material, and negative findings should be interpreted with caution.

A larger cohort is warranted for CCC to explore the associations between miRNAs and survival. However, miR-202-3p and miR-1281 were found to be associated with RD in CCC, although this could not be adjusted for stage due to the small series.

We further mapped IPA based interactions between differentially expressed mRNAs and miRNAs in HGSC. Unfortunately, global mRNA expression analysis of CCC was not available. The vast majority of these interacting RNAs has previously been associated with cancer and cancer-related functions, and may represent important key molecular pathways in HGSC. Moreover, differentially expressed miRNAs in HGSC were linked to overexpressed mRNAs in a molecular pathway for HGSC. In this latter IPA analysis, both over- and underexpressed miRNAs were included. The functional association between an overexpressed miRNA and overexpressed miRNA targets, if any, may be indirect or be due to compensatory mechanisms. For example, VEGFA, which we previously found to be overexpressed and associated with PFS in HGSC [29], is a target of miR-200c-3p. A possible explanation for interaction, in spite of both being overexpressed, may be due to adaptive mechanisms leading to overexpression of miR-200c-3p, in an attempt to reduce VEGFA and consequently carcinogenesis. However, an interaction resulting in activation of VEGFA expression can not be ruled out [50-52]. The identified interactions are IPA based, and should be experimentally evaluated in HGSC.

OSE was in this study used as control material, since OC is presumed to originate in the OSE [53]. However, an alternative origin of a subset of OC has recently been proposed, suggesting implanted epithelial cells of the fallopian tube and endometrium in the ovary as an origin for HGSC and CCC, respectively [3]. The basis for this proposed model are findings of tubal dysplasia and tubal intraepithelial carcinoma (TIC) in women predisposed to [54,55] or operated for [56] HGSC, as well as a molecular resemblance of TIC to HGSC [57-59]. However, since a direct transition from lesions in the Fallopian tube to OC has still not been demonstrated, OSE may still be the origin for OC. Interestingly, a common embryological origin of fimbrial epithelium and OSE has been hypothesized [60], which may explain a similar predisposition for the development of tubal and ovarian cancer.

## Conclusions

Several miRNAs significantly differentially expressed between HGSC, CCC and OSE were identified through global miRNA expression profiling and RT-qPCR validation analysis, suggesting a role for these miRNAs in OC. The differences emphasize the biological distinctiveness of these OC subgroups. Highly overexpressed miRNAs including miR-205-5p in HGSC and members of the miR-200 family in HGSC and CCC target EMT drivers, and may be important in OC progression. Overexpression of miR-182-5p and miR-200a-5p and underexpression of miR-383 was also found in HGSC and CCC. Some miRNAs separating CCC from HGSC were also identified, including miR-509-3-5p, miR-509-5p, miR-509-3p and miR-510. miR-200c-3p, the most significantly differentially expressed miRNA in both HGSC and CCC, was found to be associated with PFS and OS in HGSC, representing a potential prognostic marker for HGSC. In HGSC, several interacting differentially expressed miRNAs and mRNAs were mapped, but need to be experimentally verified. The identified miRNAs should be explored in future studies as candidate biomarkers and therapeutic targets.

## Competing interests

The authors declare that they have no competing interests.

## Authors’ contributions

BVE conceived and designed the experiments, performed patient recruitment, tissue sampling and collection of clinical data, performed experiments and the Ingenuity pathway analyses, analyzed the data, performed statistical analyses and wrote the paper. OKO designed the experiments, performed experiments, analyzed the data and performed statistical analyses. KBFH designed the experiments and analyzed the data. BB performed experiments and analyzed the data. LS performed statistical analyses. AS established and was responsible for the research biobank that provided most patient recruitment. KMG designed the experiments and analyzed the data. BD designed the experiments, performed tissue sampling, reviewed the histological material, was consultant in pathology and analyzed the data. All authors discussed the results, contributed to preparation of the manuscript and approved the final manuscript version.

## Pre-publication history

The pre-publication history for this paper can be accessed here:

http://www.biomedcentral.com/1471-2407/14/80/prepub

## Supplementary Material

Additional file 1 FC- and p-values for 78 differentially expressed (ANOVA, FDR < 0.01%) miRNAs between HGSC, CCC and OSE. Click here for file

Additional file 2 FC values for IPA based experimentally observed mRNA targets of differentially expressed miRNAs in HGSC vs. OSE. Click here for file

Additional file 3 **Context + scores for predicted interactions of differentially expressed (FC ≥ ±10) miRNAs and mRNAs in HGSC.** miRNAs and predicted mRNA targets are shown in columns. Number of conserved binding sites is given after each mRNA (superscript). All predicted interactions are of high predicted confidence. FC values are provided in Table 5. Click here for file
