# Supplementary material for: Global miRNA expression analysis of serous and clear cell ovarian carcinomas identifies differentially expressed miRNAs including miR-200c-3p as a prognostic marker
Source: BMC Cancer. 2014 Feb 11;14:80. doi: 10.1186/1471-2407-14-80 (PMC3928323; doi:10.1186/1471-2407-14-80)
Supplement: Additional file 2 — FC values for IPA based experimentally observed mRNA targets of differentially expressed miRNAs in HGSC vs. OSE. [file 1471-2407-14-80-S2.pdf]

Additional file 2. FC values for IPA based experimentally observed mRNA targets of differentially expressed miRNAs in HGSC vs. OSE.

**mRNAs**	**FC values**	**mRNAs**	**FC values**	**mRNAs**	**FC values**
ADCY6	1.1	FHOD1	1.1	PIK3CA	1.6
BAP1	1.4	FNDC3A	-2.5	PLAG1	1.0
BCL2L14	1.8	FOXO1	-4.3	PLCG1	-1.1
CARD11	1.6	FOXO3	1.0	PPM1F	1.1
CASP10	1.8	GADD45G	-5.5	PRKCE	-1.1
CASP2	2.4	IGF1R	-1.6	PTPN13	-1.7
CD274	1.2	INPPL1	2.6	PTPRD	-3.6
CDH1	6.0	JAG1	1.1	RARG	1.5
CDH4	-1.2	KLHL20	1.0	RERE	1.3
CDK6	-1.1	LRP6	1.1	RPS6KB1	-1.5
CDKN1B	-1.7	MALAT1	-1.6	SOS1	-1.3
CLDN15	-2.5	MAP2K1	1.3	SPDEF	2.2
CLOCK	1.0	MAP2K4	-1.2	TGFB2	-2.0
COL11A2	1.2	MARCKS	-1.2	TUBB	1.6
COL4A4	-2.9	MED1	-1.2	VEGFA	5.7
CTBP2	1.5	MITF	-3.0	VIM	-2.5
CTNNB1	-1.3	MTSS1	-1.4	VWF	2.3
CYP1B1	2.9	NCAM1	-3.1	WASF3	-4.6
ELMO2	1.3	NFASC	-3.8	WDR37	-1.0
ERBB2IP	-1.0	NFIA	-1.8	ZEB1	-3.8
ERBB3	20.9	NLGN2	1.1	ZEB2	-5.7
ERRFI1	-3.1	NTRK3	1.1	ZFPM2	-4.5
FGFR1	-1.4	PGF	1.3		

The FC (Fold change) values are based on global gene expression analysis [29]. mRNAs with signal values $<2^5$ are excluded. IPA: Ingenuity Pathway Analysis. HGSC: High-grade serous ovarian carcinoma. OSE: ovarian surface epithelium.
