# Supplementary figures and images for: Global miRNA expression analysis of serous and clear cell ovarian carcinomas identifies differentially expressed miRNAs including miR-200c-3p as a prognostic marker
Source: BMC Cancer. 2014 Feb 11;14:80. doi: 10.1186/1471-2407-14-80 (PMC3928323; doi:10.1186/1471-2407-14-80)

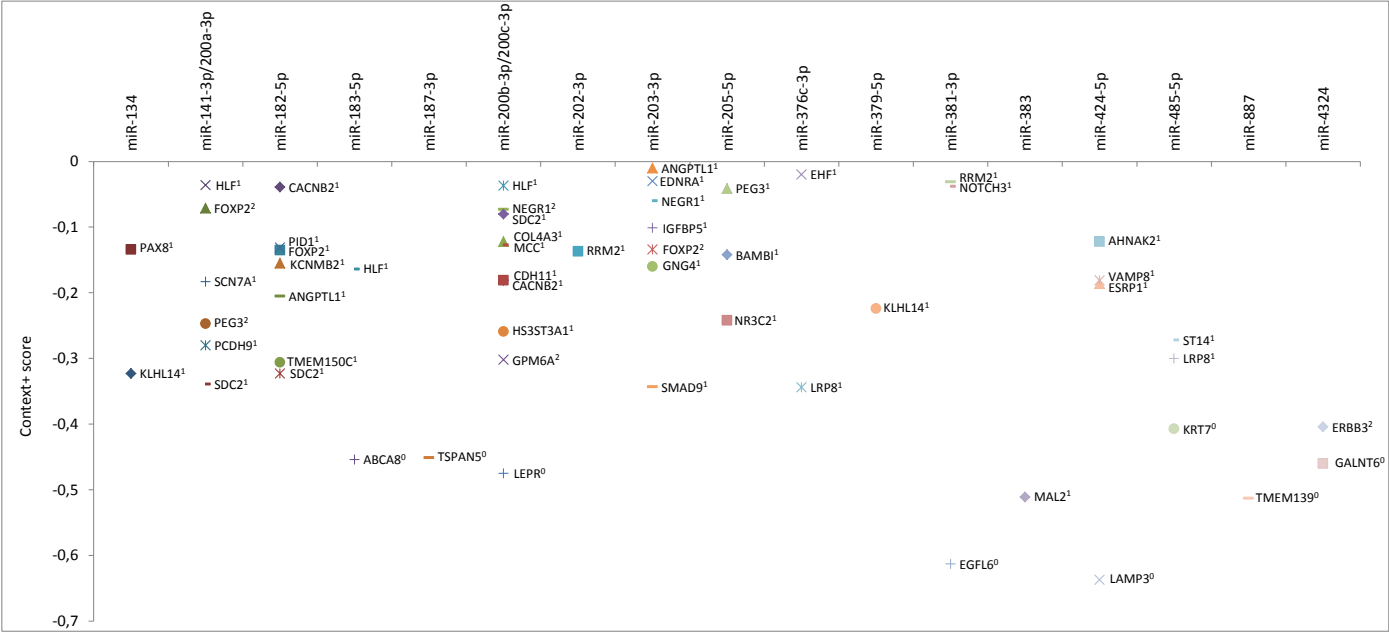

Supplement: Additional file 3 — Context + scores for predicted interactions of differentially expressed (FC ≥ ±10) miRNAs and mRNAs in HGSC. miRNAs and predicted mRNA targets are shown in columns. Number of conserved binding sites is given after each mRNA (superscript). All predicted interactions are of high predicted confidence. FC values are provided in Table 5. [file 1471-2407-14-80-S3.pdf]
